# Sustainability in food-based dietary guidelines: a review of recommendations around meat and dairy consumption and their visual representation

**DOI:** 10.1080/07853890.2025.2470252

**Published:** 2025-03-07

**Authors:** Maddie Sinclair, Emilie Combet, Tess Davis, Esther K. Papies

**Affiliations:** ^a^ Social and Public Health Sciences Unit, School of Health and Wellbeing, University of Glasgow, Glasgow, UK; ^b^ School of Medicine, College of Medical, Veterinary & Life Sciences, University of Glasgow, Glasgow, UK

**Keywords:** Sustainable diets, food-based dietary guidelines, plant-based diets, meat consumption, dairy consumption

## Abstract

**Background:**

The transition away from high meat and dairy consumption and towards more plant-based diets is vital for environmental sustainability targets, including reducing greenhouse gas (GHG) emissions and land use associated with food. Food-based dietary guidelines (FBDG) communicate nutrition information to a country’s general public. However, it is unknown how different countries’ FBDG communicate reducing meat and dairy intake in the context of sustainability.

**Methods:**

To address this gap, we reviewed global consumer-facing FBDG (*n* = 58) in middle- and high-income countries to examine recommendations and information around meat and dairy consumption, and to explore the pictorial representation of these foods.

**Results:**

Few countries used a sustainability argument to recommend reducing meat (6/58) or dairy consumption (2/58). The proportion of dairy images within food guides was consistently higher than meat images. Some countries’ guidelines are starting to consider meat intake in the context of sustainability and implementing meat reduction recommendations. However, this is not the case for dairy, potentially due to complex nutritional implications.

**Conclusion:**

Overall, our review shows that very few countries recommend reducing either meat or dairy consumption. To reduce the environmental impact of food systems, clearer guidelines backed by current evidence are needed, which provide justification behind recommendations, actionable advice for how to meet the recommendations, and appropriate quantified food targets or limits. This well-rounded advice is imperative to empower citizens to take action on their dietary habits, to reduce global meat and dairy consumption and replace these with more sustainable alternatives for human and planetary health.

## Introduction

Food systems worldwide contribute between a quarter [1] and a third [2] of global greenhouse gas (GHG) emissions. Cattle products, mainly consisting of meat and dairy, make up 52% of global emissions from agriculture [3]. Whilst the environmental impact of these foods varies across a wide range of farming practices, meat from ruminant animals (e.g. beef, lamb), and dairy products, especially cheese, are amongst the most resource-intensive foods with the highest environmental impact [1]. Beef from a beef herd at the lowest end of the range for its environmental impact (i.e. the 10th percentile) still emits 10 times more GHG than the mean GHG emissions of tofu and requires over 19 times more land [1]. With regards to dairy [1], also found that the 10th percentile of GHG associated with cheese is 1.5 times higher than the mean GHG emissions associated with tofu, and double the GHG emissions associated with tofu for land use. Dairy milk is consistently more environmentally impactful than any plant-based alternative, with GHG emissions being at least 3 times higher, land use at least 11 times higher, and freshwater use at least 1.5 times higher [1].

Whilst the figures cited above may not always reflect direct dietary substitutes, they demonstrate the environmental impact of consuming meat and dairy, especially cheese. People in high-income countries consume the most meat and dairy [4]. Due to this consumption, the GHG emissions of diets in these countries exceed environmental limits of planetary boundaries, including GHG emissions, land use and bluewater use. This would still be the case, even if these were in line with national and global dietary guidelines, particularly in North America and Europe [5].

Therefore, there would be substantial environmental benefits to populations reducing the consumption of animal products, particularly meat and dairy, from habitual consumption levels. This would be true for a uniform decrease in intake by all, or a more substantial decrease by the highest consumers, altogether shifting the median intake downward. The environmental benefits of these reductions include reducing land use, GHG emissions, and water usage [6]. Additionally, this would mitigate biodiversity loss, of which livestock production is the largely responsible [7].

When reducing meat and dairy consumption, implications for dietary change need to be considered. Dairy foods are nutrient-dense, affordable, accessible and acceptable. Therefore, they can play an important role in meeting nutritional requirements, a particularly important consideration for vulnerable populations such as low-income households [8], and for groups at risk of deficiencies, such as young women [9]. However, current climate change targets include reducing meat and dairy within Western diets, such as the UK Climate Change Committee recommendations to reduce meat and dairy consumption by 20% by 2030, and to reduce meat consumption by 35% by 2050 [10] Despite socioeconomic inequalities within high-income countries, the average dairy intake in high-income countries needs to be reduced for environmental sustainability [11].

Food-based dietary guidelines should also be considered in the context that the population-wide dietary consequences of Western diets tend to be related to overconsumption, with 1 in 8 people worldwide living with obesity [12]. Part of this is related to highly processed foods, particularly ultra-processed foods, which are currently at the centre of a complex debate. Whilst this is typically focused on health, there is also concern about their environmental impacts, particularly when they contain meat and when these foods form a substantial proportion of the diet. Overall, the multiple implications of proposed dietary shifts must be considered, including health and economic impacts.

It is unclear to what degree the need for dietary change away from animal-based foods and towards plant-based foods is communicated to citizens (i.e. residents of a particular country), through dietary guidelines, which are the primary tool governments and health authorities use to communicate with citizens about food recommendations. To address this gap, we examined what information consumer-facing food-based dietary guidelines (FBDG) include about reducing meat and dairy intake. We analysed both recommendations to reduce meat and dairy intake in written guidelines in the context of environmental sustainability, and the pictorial representation of meat and dairy in guidelines.

### Food-based dietary guidelines

FBDG communicate nutritional information to the public, translating the available scientific evidence into accessible and actionable advice. FBDG contain written recommendations, often accompanied by a food guide, i.e. a graphical representation of guidelines to facilitate communication with citizens [13]. They are often supplemented with background documents, such as a more comprehensive set of guidelines aimed at health professionals, documents outlining the development process of guidelines, and documents containing the scientific evidence that forms the basis of guidelines. According to the Food and Agriculture Organization of the United Nations (FAO), dietary guidelines should be ‘short, simple, clear and memorable’ [14], and preference for short messages in guidelines has been echoed in consumer research, e.g [15]. Consumer-facing dietary guidance must not overburden citizens with overwhelming detail; being concise is critical to the real-life use of guidelines [16]. A recent review of communication strategies of the UK Eatwell Guide reinforced this, concluding that language within dietary guidelines should be short, simple, specific and easy to understand [17].

Overall, FBDG provide advice and expectations around consumption, and they are often specific to each country to reflect culturally specific dietary patterns [18]. Almost 100 countries globally have implemented FBDG. Their widespread use demonstrates their potential as a public health policy tool to regularly provide current information around dietary patterns, such as sustainable diets, to support public health whilst considering other aspects of multi-faced food systems [19,20]. It has been argued that failure to include environmental sustainability in future guidelines will result in dietary guidelines that do not communicate fully about key implications of food choices that are of interest to many citizens, namely individual physical health, as well as planetary health and sustainability [21] Furthermore, citizens across the globe are concerned about climate change, and are strongly in favour of political action to mitigate this, as well as supporting pro-climate norms [22]. Therefore, we anticipate that citizens will increasingly expect governments to incorporate sustainability into areas of communication where appropriate.

Most research into FBDGs so far has focused on health-related content. Systematic reviews have examined health-related key messages in guidelines [20] and health messages and visual representations [23,24]. Additionally, some research into FBDGs has focused on environmental sustainability considerations. For example, environmental sustainability-related content [13], and alignment with guidelines to the Sustainable Healthy Diets Guiding Principles as outlined by the World Health Organisation and the FAO [25,26]. Additionally, a recent qualitative review examined visual representations of dietary and sociocultural norms in guidelines [18] which found that certain imagery (e.g. cultural symbols) and tools (e.g. the use of proportionality) are used to guide individuals towards healthy diets. In contrast [27,] found that when focusing on including plant-based substitutes and sustainability within guidelines, many lacked sufficient information.

Two modelling studies have assessed the potential implications of existing guidelines on future health and planetary outcomes [5,28]. They found that following current global dietary guidelines, focused on healthy diets, would be insufficient for environmental sustainability considerations, including reducing GHG emissions, water use and land use from food production. Furthermore, a more recent modelling study quantified recommendations from 85 countries’ dietary guidelines in comparison to global dietary guidelines from the WHO and the EAT-Lancet Commission [5]. This showed that whilst adopting the dietary patterns outlined in these guidelines would improve health outcomes in two thirds of cases, most would not improve environmental sustainability such as reducing GHG emissions and land use. This was notably in part due to weak or insufficient recommendations for meat and dairy consumption, with many countries even recommending increasing dairy intake. In sum, evidence so far suggests that environmental sustainability is not sufficiently considered or covered within FBDG, especially not in comparison to health-related recommendations [25,26], often appearing secondary to core information and not used in the justification for recommended quantities [29].

Research has yet to focus directly on environmental sustainability considerations in consumer-facing guidelines. For example, Martini et al. [26] focused on ‘key messages’ in guidelines, yet whether these came from consumer-facing guidelines or background documentation was unclear. Furthermore, while James-Martin et al. [25] distinguished between background and consumer-facing guidelines, some guidelines identified as consumer-facing were over 100 pages long and lacked a food guide or supporting imagery, as well as summarising information. Therefore, the current review sets out well-defined criteria for consumer-facing guidelines to assess the information that citizens will most likely encounter, use and understand.

### Visual representations within food-based dietary guidelines

The visual representation of food plays a key role in nutrition communication, as it allows for quick, effortless, and practical message processing. However, visual representations have been scarcely explored within dietary guidelines. Some research has examined food guides (i.e. the visual representation of guidelines), categorising the types of visual food guides, e.g. pyramids and plates [20,30], investigating citizen preferences for different types of visual food guides [31], or analysing imagery in guidelines from a sociocultural perspective [18]. Some of this research identified the proportionality of images within food guides as a dimension of interest. Specifically, proportionality has been studied through analysis of plate format food guides [30] and the representation of normative portion sizes and daily intake [18]. Therefore, the pictorial representation of images in guidelines may be argued to represent national or cultural food norms, or may be interpreted by citizens as such, and the foods pictured can communicate information about both individual food items and nationally relevant dishes [32]. In the current review, we build upon previous research to specifically investigate the proportion of meat and dairy images relative to other images within a country’s food guide, both within a food group (e.g. ‘protein-rich foods’) and in relation to all images within a guide. The aim of this is to assess the implicit recommendations for their consumption.

### The current research

The current rapid review investigates whether consumer-facing FBDGs recommend reducing meat and dairy intake in the context of sustainability. We aimed to understand the information and recommendations presented in dietary guidelines regarding meat and dairy consumption and whether they were justified by sustainability arguments. This also included understanding how meat and dairy were portrayed visually in food guides, regarding the number of relevant images per food group.

Using two sets of keywords, we systematically searched the text of the guidelines for statements about environmental sustainability, meat consumption, and dairy consumption. Combined, this enabled us to see if guidelines contained general information about sustainability and diets, what their recommendations around meat and dairy consumption were, and if recommendations were motivated by sustainability. Furthermore, we analysed the proportion of pictures of meat and dairy within food guides, both within food groups and as a proportion of images overall within the food guide.

## Methods

We used the FAO database to retrieve guidelines for countries classified as middle or high-income by the World Bank, which have higher proportion of GHG emissions [33], and available data for the country’s food system GHG emissions using FAO data. For middle or high-income countries that did not have guidelines on the FAO database or if guidelines were otherwise irretrievable, a structured Google search was used as outlined in Appendix 1. We deemed guidelines as eligible for the review if they were in English or searchable as a PDF in another language. Guidelines existing in an image format only were excluded. MS carried out the first search in October 2022, with an updated search in June 2023 (MS), when the second reviewer (TD) screened guidelines for eligibility and collected and analysed data from the guidelines.

### Search strategy

#### Dietary guidelines search

We screened selected guidelines for those that were consumer-facing, for which we set out clear criteria; to our knowledge, no previous research has done this. Key criteria were that guidelines were 1) summarised with headed sections or numbers, 2) contained more than a plain-text document, and 3) were explicitly consumer-facing in language, i.e. directed at the general population either in the title (e.g. ‘General dietary guidelines for Koreans’), within the guidelines, or on the relevant website. If guidelines contained only a food guide (a graphical representation of guidelines), without written guidelines to accompany or as part of the guide, they were excluded (*n* = 1). For countries whose guidelines included a food guide, this was included in the pictorial analysis. If an eligible country’s guidelines did not include a food guide, the relevant website (e.g. a health authority or government department) was searched using the specific name of the guidelines to search for an accompanying food guide. Appendix 2 outlines all potential countries and the inclusion and exclusion of countries’ FBDG down to the final sample (*n* = 58). All guidelines and appendices are stored in the OSF repository: https://osf.io/sqw48/

### Data collection

We identified eligible guidelines by using two sets of keywords. The primary set of keywords was used to identify environmental sustainability information within guidelines and were as follows: ‘Sustain*’, ‘Environment*’, ‘Climat*’ and ‘Planet*’. The secondary search terms were used to identify meat and dairy-focused information and were as follows: ‘Plant*’, ‘Animal’, ‘Meat’ and ‘Dairy’. These two searches were carried out sequentially to separate the analysis of sustainability information and meat and dairy information within guidelines. The separation of the searches enabled analysis of meat and dairy information distinctly from sustainability. We used Google Translate to translate these terms from English into the target language for guidelines not in English. We then searched the guidelines using these translated terms, translated the surrounding information into English using Google Translate, and extracted the relevant statements. From the keyword search, we extracted statements containing the words verbatim. We did not include titles and subheadings within the guidelines.

### Data analysis

We categorised statements from the primary keyword search as to whether they were generic or all-encompassing in relation to sustainability and diets, if they focused on meat intake, or if they focused on dairy (and therefore included any secondary search terms). If guidelines contained only references to sustainability without mentioning dietary patterns, we excluded these. Likewise for guidelines mentioning ‘Environment’ in reference to the food environment, such as in Australia’s consumer-facing FBDG (see OSF for details).

We then screened the selected guidelines for secondary keywords and extracted statements. We categorised statements as to whether they contained explicit recommendations around meat and dairy intake, whether the recommendation was to reduce the target food consumption, or if the recommendation was neutral, i.e. mentioning the consumption of meat/dairy but without giving a specific direction for the required intake (such as the recommendation from Qatar to ‘maintain a daily consumption of skimmed or low-fat milk and dairy products’). The recommendations were then separated into those motivated by sustainability (i.e. also containing primary search term criteria) and those that were not.

Lastly, using statements and recommendations from the primary and secondary search terms, we scored guidelines using a method adapted from James-Martin et al. [25]. Each country was scored on whether they mentioned the ‘what’ (core advice), ‘why’ (why the advice is important/relevant in the context of sustainability), ‘how’ (how to meet the advice practically) and ‘quantity’ (a specific quantity of food in question to help meet the advice) in their guidelines, relating to reducing meat and dairy intake in the context of sustainability. This multi-dimensional analysis of guidelines is particularly important, as it aligns with the need for dietary guidance to include actionable advice about how and why to change behaviour [16].

Data collection and data analysis were carried out by two of the authors separately and independently (MS and TD). Any uncertainties around meeting inclusion criteria, categorisation of statement(s) as generic/meat/dairy, inclusion of statement(s) as a recommendation, categorisation of meat and dairy intake recommendations as neutral/reduce, or the definition of what/why/how/quantity scoring were discussed between MS and TD. If applicable, MS and TD reached a consensus regarding any discordance during several meetings.

### Pictorial analysis from food guides

For the countries that met the criteria for the analysis of written guidelines, we carried out a pictorial analysis for food guides within guidelines or otherwise accompanying them as outlined above. First, we counted the number of meat and dairy images in the whole guide. We then calculated the proportion of each of these in relation to the overall images within a food group (e.g. protein-rich foods or dairy), and in relation to all images overall within the food guide. We carried this out separately for images of meat and dairy. If a food guide had no distinct food groups, we did not calculate the proportion of meat/dairy images within a food group. We did not consider the relative size of images. If meat/dairy images occurred over several categories, we took an average across groups. The proportions were initially calculated as fractions (e.g. 1/5 for 1 meat or dairy image in comparison to a total of 5 images in the overall group). They are expressed as percentages for ease of comparison and interpretation; original proportions as fractions can be found in Appendix 5.

## Results

### Analysis of written guidelines

To analyse written information in guidelines, we identified 73 countries with dietary guidelines classified as middle or high-income countries. 58 of those countries had guidelines available in English or otherwise searchable if in another language, and we identified them as having consumer-facing guidelines. A complete list of countries can be found in Appendix 2.

**Figure 1.**
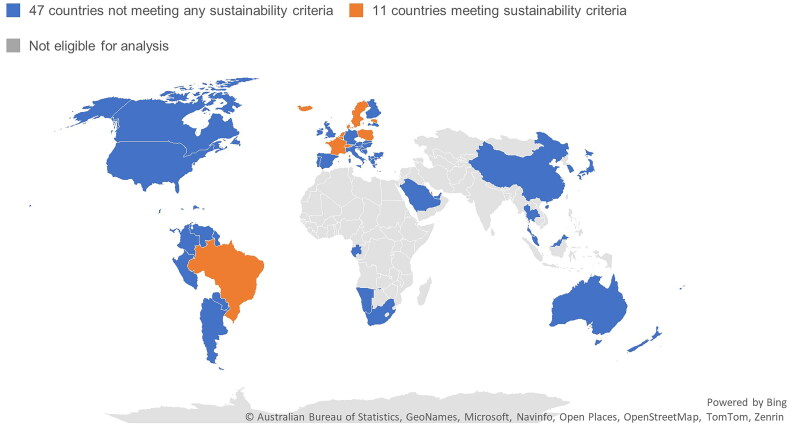
The proportion of eligible countries’ dietary guidelines (n = 58) that do and do not contain at least one of the diet-related sustainability keywords; i.e. countries meeting initial sustainability criteria.

Of the 58 eligible countries, 11 countries’ dietary guidelines contained at least one of the primary sustainability keywords related to diets (‘Sustain*’, ‘Environment*’, ‘Climat*’ and ‘Planet*’). 81% (*N* = 47) of eligible countries failed to mention sustainability considerations for diet (Figure 1).

We extracted statements containing our selected sustainability keywords and categorised them into ‘generic’, ‘related to meat intake’, or ‘related to dairy intake’ (Table 1). A full table of all extracted statements, how they meet primary and secondary search criteria, and categorisation into generic/meat/dairy information can be found in Appendix 3. Most guidelines meeting the sustainability criteria contained generic information about sustainability and diet and information about meat intake and sustainability. Only Denmark, Estonia and Sweden provided information about dairy and sustainability (Table 1).

**Table 1.** Countries’ guidelines (n = 11) with information that contains at least one of the meat and dairy-related sustainability keywords, categorised into generic, meat information, and dairy information (displayed alphabetically).

Country	Generic sustainability-related information	Meat information	Dairy information
Belgium	**✓**	**✓**	**X**
Brazil	**✓**	**X**	**X**
Denmark	**✓**	**✓**	**✓**
Estonia	**X**	**✓**	**✓**
France	**X**	**✓**	**X**
Iceland	**✓**	**X**	**X**
The Netherlands	**✓**	**✓**	**X**
Poland	**X**	**✓**	**X**
Qatar	**✓**	**X**	**X**
Sweden	**✓**	**✓**	**✓**
Switzerland	**✓**	**X**	**X**

Table 2 shows examples of extracted statements using the sustainability keywords for each country’s guidelines. A full list of all extracted statements can be found in Appendix 3. We classified statements as generic if they mentioned sustainability considerations for diets, but were not explicit in giving direction, i.e. increasing or reducing consumption. If meat or dairy were explicitly mentioned in conjunction with sustainability, we classified these as ‘meat information’ or ‘dairy information’ as appropriate. Guidelines that provided generic information about sustainability and diet contained information about GHG emissions, air quality, soil quality, land and water use and biodiversity. For meat information, this included comparing environmental metrics across animal foods, between animal and plant-based foods, and explaining the environmental impact of food required for animals. For dairy, this also included environmental metrics for dairy and the link between cows and methane.

**Table 2.** Sustainability information from guidelines, referring to generic, meat or dairy intake: (displayed alphabetically by country name).

Country	Generic sustainability-related information	Meat information	Dairy information
Belgium^b^	‘The production, processing and distribution of food has an impact on the climate, air quality, soil quality, land and water use and biodiversity. At the moment, the environmental impact of our diets is higher than what our planet can bear, and we are running into problems’	‘Plant-based foods generally have a lower environmental impact compared to animal-based foods: lower greenhouse gas emissions and less water and land use. Legumes have a very low environmental impact compared to meat and, like whole grains, are interesting as a vegetable protein source’	**X**
Brazil	‘Natural or minimally processed foods, in great variety, and mainly of plant origin, are the basis for diets that are nutritionally balanced, delicious, culturally appropriate, and supportive of socially and environmentally sustainable food systems’^a^	**X**	**X**
Denmark	‘What we eat and drink affects both our health and the climate. Therefore, the Official Dietary Guidelines show how you can eat a both healthy and climate-friendly diet’	‘Cutting down on meat also benefits the climate. This applies to all types of meat, and in particular beef and lamb, which are among the foods with the highest climate footprint. Poultry, pork and eggs have a significantly lower impact on the climate than beef and lamb’	‘A high intake of dairy products leads to increased climate impact’
Estonia^b^	**X**	‘Meat is one of the foods with the biggest negative environmental impact, as its production requires a lot of water, land (especially for growing fodder) and produces a large amount of greenhouse gases. Therefore, reducing the amount of meat on the plate can significantly reduce the environmental footprint of food’	‘Milk is a nutrient-rich food, but it has a high environmental impact. The production of milk and dairy products requires a lot of water, land and energy and produces a large amount of greenhouse gases’
France	**X**	‘More pulses and less meat, it’s good for the environment. Of all foods, it’s meat which has the biggest impact on the climate’	**X**
Iceland^b^	‘Most Icelanders would benefit from increasing their consumption of plant-based food, e.g. on vegetables, fruits, berries, nuts, beans, lentils, seeds and whole grains. Increased consumption of plant products and reduced consumption of animal products will also help to limit the emission of greenhouse gases and thus protect the environment’^a^	**X**	**X**
The Netherlands	‘Recommended daily amounts in the Wheel of Five takes into account the environmental impact involved, by setting maximum limits for animal products’	‘We encourage people to adopt a dietary pattern with less meat but with more pulses and nuts. We advise consumers on how to make sustainable choices’ ^a^	**X**
Poland^b^	**X**	‘For health and the environment, replacing meat with protein products of plant origin, i.e. pulses (beans, chickpeas, soybeans, peas, lentils, broad beans) and nuts, as well as fish and eggs’^a^	**X**
Qatar	‘Eat healthy while protecting the environment: emphasise a plant-based diet, including vegetables, fruit, whole grain cereals, legumes, nuts and seeds’^a^	**X**	**X**
Sweden	‘We’ll give you advice and handy tips here to make it easier for you to adopt successful eating habits that are sustainable for both your health and the environment’	‘Of all foods, meat has the greatest impact on our climate and environment. This is why it’s important for us to cut back on meat and be careful about what meat we do choose to eat. Poultry has the smallest impact on our climate, followed by pork. Beef and lamb have the greatest impact…’	‘Dairy products come from cows, which release methane gas. This is bad for the environment, so it’s a good idea not to consume too much cheese or other dairy products’
Switzerland	‘Sustainable eating habits comprise: preference of plant-based foods, foods that are environment- and animal-friendly, seasonal, regional and in compliance with fair trade principles, avoiding food waste’^a^	**X**	**X**

^a^The statement listed in this cell is the only example in this category for this country’s guidelines.

^b^Extracts from this country’s guidelines have been translated into English.

#### Recommendations to reduce meat and dairy intake

**Figure 2.**
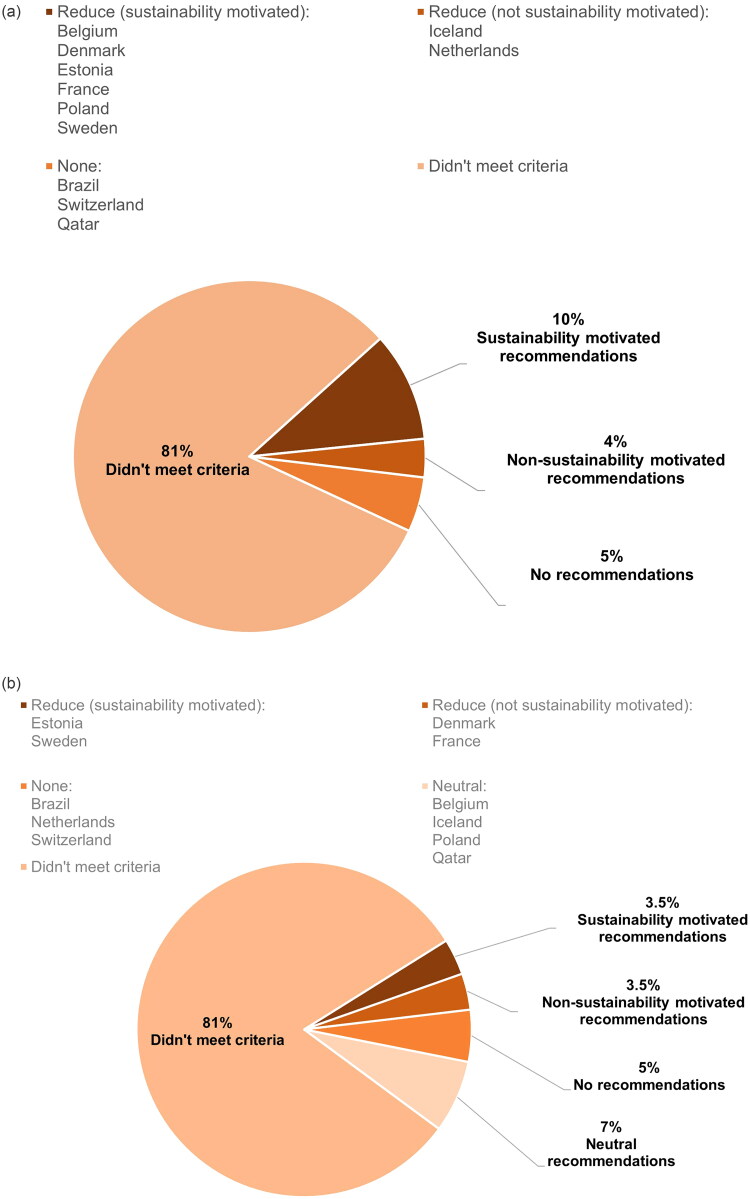
Categorisation of recommendations in guidelines for meat (A) or dairy (B) for all eligible countries (n = 58), into none, neutral (where appropriate), reduce, and recommendations to reduce, motivated by sustainability or not motivated by sustainability.

From the secondary keyword search, we analysed which countries’ guidelines recommended reducing meat or dairy consumption. Of the 11 countries meeting the sustainability criteria, six countries recommended to reduce meat consumption with sustainability motivations (10% of all eligible countries; Figure 2a) and two countries recommended to reduce dairy consumption with sustainability motivations (3.5% of all eligible countries; Figure 2b). We only found neutral recommendations for dairy consumption, not for meat consumption. For example, Denmark’s recommendation that ‘About 250 ml milk or dairy products a day is adequate when eating a plant-rich and varied diet’. A full list of all extracted statements for recommendations to meat and dairy consumption can be found in Appendix 4.

#### Scoring of guidelines for actionable sustainability advice

Lastly, we scored all guidelines meeting the primary search criteria against a brief set of criteria (‘what’, ‘when’, ‘how’, ‘why’) to examine the depth and practicality of guidance around meat and dairy consumption, based on [25]. All 11 countries’ guidelines met the ‘what’ criteria, indicating that they provided information about meat or dairy consumption and sustainability. Seven countries’ guidelines met the ‘quantity’ criterion. However, it is worth noting that this was never explicitly written in the context of sustainability.

Belgium, France and Sweden were the only countries to meet all four criteria; Table 3 shows a full breakdown of how many criteria each country’s guidelines met out of a possible four. Table 4 contains example statements for each country for each criterion they met, where possible.

**Table 3.** Scoring of the guidelines (n = 11) in line with the four dimensions of the practicality of dietary recommendations, adapted from (25] (displayed alphabetically).

Country	What	Why	How	Quantity
Belgium	✓	✓	✓	✓
Brazil	✓	X	X	X
Denmark	✓	X	✓	✓
Estonia	✓	✓	X	✓
France	✓	✓	✓	✓
Iceland	✓	✓	X	✓
The Netherlands	✓	X	✓	X
Poland	✓	X	✓	✓
Qatar	✓	X	X	X
Sweden	✓	✓	✓	✓
Switzerland	✓	X	X	X

**Table 4.** Information and recommendations from guidelines (n = 11) and how they meet the four dimensions of the practicality of dietary recommendations, adapted from (25] (displayed alphabetically).

Country	What (core advice/information)	Why (why is the advice important in the context of sustainability)	How (how to meet the advice)	Quantity (specific quantity of food(s) to eat to meet the advice)
Belgium	‘Reducing the current consumption of meat and other food of a possible origin, such as cheese, provides major environmental benefits’	‘Plant-based foods generally have a lower environmental impact compared to animal-based foods: lower greenhouse gas emissions and less water and land use. Legumes have a very low environmental impact compared to meat and, like whole grains, are interesting as a vegetable protein source’	‘Legumes, whole grains and nuts are healthy plant protein sources full of beneficial nutrients for your body… And also tasty and often quick and easy to prepare: a chili sin carne (a chili without meat) is on the table in no time’	‘The Superior Health Council recommends that adults consume no more than 300 g of red meat and no more than 30 g of processed meat per week’
Brazil	‘Natural or minimally processed foods, in great variety, and mainly of plant origin, are the basis for diets that are nutritionally balanced, delicious, culturally appropriate, and supportive of socially and environmentally sustainable food systems’.^a^	X	X	X
Denmark	‘A high intake of dairy products leads to increased climate impact’.	X	‘Introduce meat-free days and cut down on meat in your meals’	‘Cut down on meat. About 350g of meat is adequate when eating a plant-rich and varied diet’
Estonia	‘How much and what kind of meat products are consumed not only has a significant impact on health but also on the environment. Therefore, limit yourself to the recommended amounts when eating meat’	‘Meat is one of the foods with the biggest negative environmental impact, as its production requires a lot of water, land (especially for growing fodder) and produces a large amount of greenhouse gases’	X	‘300-400g of poultry per week and 100g of red meat per week’
France	‘More pulses and less meat are what’s good for the environment. Of all foods, it’s meat which has the biggest impact on the climate’	‘Pulses don’t need fertiliser, they naturally enrich the soil and consume less water’^a^	‘If you consume more pulses…when they are combined with grains…they can even replace meat or poultry’^a^	‘Choose poultry and limit other meat (pork, beef, veal, mutton, lamb, offal) to 500g per week’^a^
Iceland	‘Most Icelanders would benefit from increasing their consumption of plant-based food, e.g. on vegetables, fruits, berries, nuts, beans, lentils, seeds and whole grains’	‘Increased consumption of plant-products and reduced consumption of animal products will also help to limit the emission of greenhouse gases and thus protect the environment’	X	‘Limit consumption of red meat to 500 g per week’^a^
The Netherlands	‘The Wheel of Five takes sustainability into account. For instance, we provide maximum limits for the use of animal products such as meat (including red meat), milk products and fish’	X	‘We encourage people to adopt a dietary pattern with less meat but with more pulses and nuts’^a^	
Poland	‘For health and the environment, replace meat with plant-based protein products, i.e. seeds, legumes (beans, chickpeas, soybeans, peas, lentils, broad beans) and nuts, as well as fish and eggs’^a^	X	‘For health and the environment, replace meat with plant-based protein products, i.e. seeds, legumes (beans, chickpeas, soybeans, peas, lentils, broad beans) and nuts, as well as fish and eggs’^a^	‘Do not eat more than 500 g of red meat and processed meats (cold meats, sausages) per week’^a^
Qatar	‘Eat healthy while protecting the environment: emphasise a plant-based diet, including vegetables, fruit, whole grain cereals, legumes, nuts and seeds’ ^a^	X	X	X
Sweden	‘Of all foods, meat has the greatest impact on our climate and environment’.	‘Dairy products come from cows, which release methane gas. This is bad for thenvironment, so it’s a good idea not to consume too much cheese or other dairy products’	‘Focus more on vegetarian foods and eggs, and sometimes fish or poultry’	‘Eat less red and processed meat, no more than 500 grams a week’^a^
Switzerland	‘Sustainable eating habits comprise: preference of plant-based foods, foods that are environment- and animal-friendly…’^a^	X	X	X

^a^The statement listed in this cell is the only example in this category for this country’s guidelines.

^b^Extracts from this country’s guidelines have been translated into English.

For countries with reductions of meat or dairy intake not motivated by sustainability, it is interesting to look at what their guidelines did contain related to sustainability. This is best done by examining the information about diet and sustainability provided across guidelines, as opposed to the recommendations to reduce meat and dairy intake. Denmark, France, Iceland and the Netherlands all had recommendations to reduce meat or dairy intake not motivated by sustainability. Iceland and the Netherlands had such recommendations for meat intake, and both had generic information about increasing the consumption of plant-based foods, including pulses and nuts, and decreasing the consumption of animal products. The Netherlands’ guidelines were more specific about reducing meat intake (‘We encourage people to adopt a dietary pattern with less meat but with more pulses and nuts’, Table 4); however, they did not provide an explicit recommendation regarding the level of intake. Denmark and France recommended reducing dairy intake without sustainability motivation. Regarding the information, Denmark outlined, ‘A high intake of dairy products leads to increased climate impact’ (Table 4), but France had no such information. The delineations between information and recommendations (as seen throughout, e.g. in Figure 2), highlight that some countries’ guidelines do not include explicit recommendations to reduce meat or dairy intake, even when there is some provision of diet-related sustainability information.

### Pictorial analysis of food guides

We included seven countries’ guidelines for the pictorial analysis. We excluded the remaining countries’ guidelines due to a lack of a food guide, or because the images within the food guide were unclear or overlapped, making it impossible to calculate proportions.

**Figure 3.**
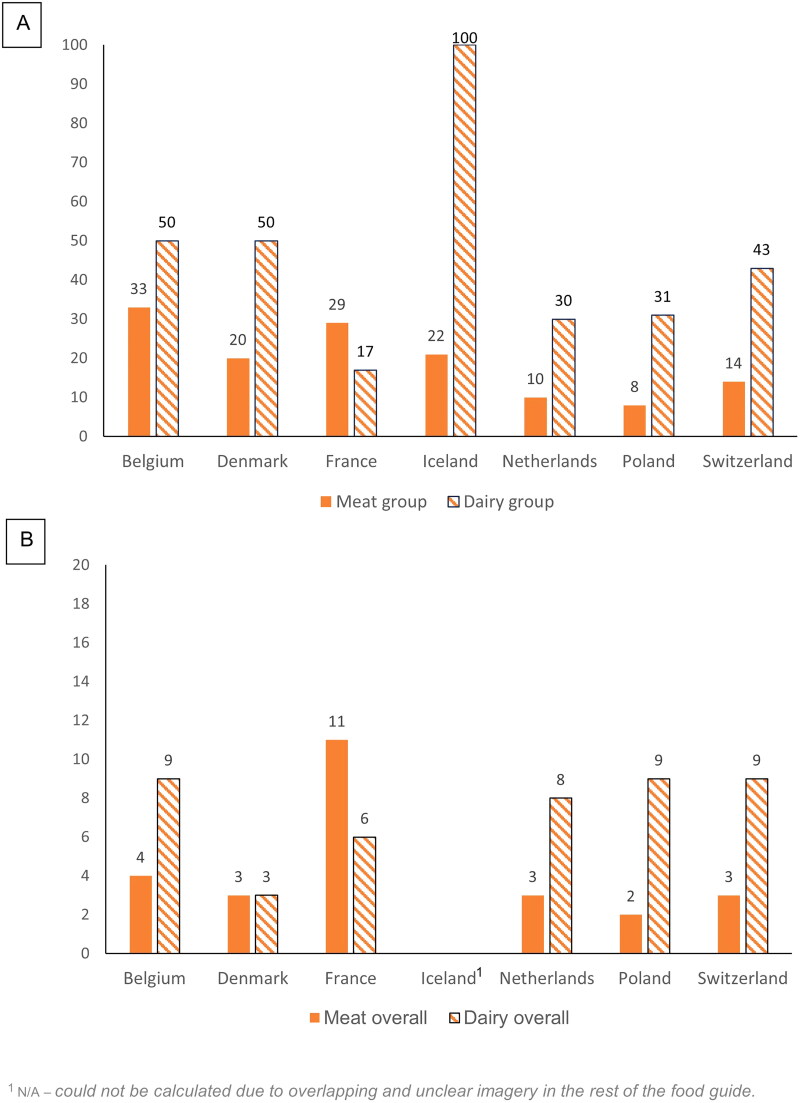
Proportion (%) of meat and dairy images out of all images within a food group (a), and out of all images within the whole food guide (B) (displayed alphabetically by country).

Figure 3 shows the proportion of meat and dairy images within food guides within a food group (A) and across all images in the food guide (B). The proportion of dairy images was higher across food groups and all images within a food guide, except in the case of France for both meat and dairy, and in the case of Denmark for dairy. The higher proportion of dairy images indicates that dairy was more prevalent than meat within food guides. This finding is in line with the analysis of written guidelines, in that more countries’ guidelines recommended reducing meat intake. There are fewer images of meat (therefore an implicit recommendation for reduction) than for dairy, for which there is a higher proportion of images.

## Discussion

### Summary of findings

We reviewed dietary guidelines for their information and recommendations around reducing meat and dairy consumption and whether these were motivated by sustainability. Furthermore, we analysed the pictorial representation of meat and dairy, specifically the proportion of meat and dairy images relative to other food images.

Out of a total of 58 eligible countries, 11 countries had guidelines that contained keywords from both the primary and secondary criteria. Of those countries, eight had guidelines that contained generic information about sustainability and diet, seven had information about sustainability and meat intake, and three had information about sustainability and dairy intake. We then examined explicit recommendations to reduce meat and dairy intake from a sustainability perspective. Six countries’ guidelines explicitly recommended reducing meat intake, while two countries recommended reducing dairy intake. Finally, only France, Belgium and Sweden met all four criteria of scoring for the practical applicability of the guidelines: the ‘what’, ‘why’, ‘how’ and ‘quantity’, to support dietary change towards more sustainable diets. These findings show that overall, only a minority of countries’ guidelines have information about sustainability considerations for diet, and equally, very few have explicit recommendations about reducing meat and dairy intake for sustainability.

When we looked at countries whose guidelines contained recommendations to reduce meat or dairy intake not motivated by sustainability, we found that the information they provided around diet and sustainability varied in detail and specificity. However, it is well established that information alone is insufficient to enact dietary change. Actionable guidance is central to supporting citizens with their dietary change, and more explicit recommendations are needed to fulfil criteria outlined in Tables 3 and 4 [17]. Recommendations need to be improved to cover why the guidance to reduce meat and dairy intake is important and relevant for sustainability, how to make the changes including appropriate food swaps to meet nutritional needs, and quantities in terms of limits and targets for animal-based and plant-based foods.

Overall, more guidelines recommend reducing meat intake, especially from a sustainability perspective, than reducing dairy intake. However, we found recommendations to reduce meat consumption from a sustainability standpoint in only 10% of all eligible countries’ guidelines. In general, sustainability and diet (in terms of reducing meat and dairy intake), are not being considered and incorporated widely across FBDG in high-income countries.

For the pictorial analysis, only seven countries’ guidelines were eligible, of which one country did not fulfil all the criteria for the full analysis. In most cases, dairy had a higher proportion of images than meat relative to other images, i) within a food group and ii) relative to all other images. Findings from the pictorial analysis are consistent with our analysis of the written guidelines, whereby explicit messaging around reducing meat intake is more apparent than that for reducing dairy intake. Likewise, the implicit (i.e. image-based) messaging for reducing meat intake inferred from the pictorial representation is more prevalent than that for dairy.

### Relating these findings to the existing literature

Overall, our findings are in line with previous research showing that dietary guidelines lack a sustainability focus [13,20, 24–27,34–36]. Our research aligns particularly closely with recent findings that guidelines lack specific actionable advice around sustainability and diet [25], especially for moving away from ruminant animal-based foods to a diet higher in plant-based foods [35]. However, there are also discrepancies between the current research and existing literature about which countries’ guidelines include information about sustainability and animal-based or plant-based diets [37.] identified that Belgium, Finland, the Netherlands and Sweden were the only EU countries to provide information about substituting plant-based foods for animal-based foods explicitly for sustainability; however, we identified three additional countries that met the ‘how’ criterion. These discrepancies are due to countries’ guidelines being included that did not meet our initial search criteria or did not contain any sustainability keywords, such as Finland [37], Bolivia, New Zealand, the Nordic Council [27], Germany, Italy, Japan, Norway and Uruguay [25]. These were included in other research due to differing criteria for inclusion, such as search terms based on the WHO guiding principles e.g [25.]. Given the key role that consumers play in the transition to sustainable diets, we decided to retain a focus on consumer-facing guidelines.

### Strengths

This review adds to the emerging literature that has examined the sustainability messaging of dietary guidelines globally [5,13, 20,23–28,34]. A key strength of this review is the explicit focus on consumer-facing guidelines that citizens are most likely to encounter and use. This resulted in our differing finding that that fewer countries’ guidelines provide information and recommendations on meat, dairy and sustainability to the public, in comparison to previous research. The focus on consumer-facing guidelines suggests that citizens do not have sufficient information about sustainability and diet, and are not receiving explicit recommendations about reducing meat and dairy consumption motivated by sustainability. This has vast implications for all facets of the food system, such as public attitudes towards food, food education and food advertising, demonstrating the pressing need to update guidelines.

Additionally, our review incorporated an analysis of the proportion of meat and dairy images relative to other images within a food group (e.g. protein-rich foods) and to all images in a food guide. To our knowledge, this analysis has not been carried out before, as existing research has focused on analysing the types of structures used for food guides, such as plates or triangles. This analysis enabled us to understand the implicit messaging through imagery (e.g. the perceived prevalence of meat images within the protein category), which complemented and reinforced our findings of the written analysis to evidence the lack of messaging around reducing meat and particularly dairy consumption.

### Limitations and future directions

In this review, we specifically focused on consumer-facing guidance only, whereas previous research has also included full guidelines and background documents. Whilst not a part of the formal analysis, we encountered background documents for certain countries due to our search strategy. We found that full guidelines and background documents, such as those from Qatar and the UK, at times included further diet-related sustainability information. Further research could compare consumer-facing guidance with full guidelines and background information, which could help to identify shortcomings. For example, some countries provide information in full guidelines or background documentation but not in consumer-facing guidelines, whereas other countries do not provide any sustainability information in any documentation. An analysis of where sustainability-related information is presented may be conducted alongside an analysis of power relationships and financial interests that may affect decision making around dietary guidelines.

We recognise that our resources and human error or biases may have resulted in erroneously identifying guidelines as consumer-facing and misinterpreting translations of the guidelines. As with other research [24,25], we translated guidelines into English where possible using Google Translate. Future research could involve stakeholder engagement, and wider international research networks to assist with searching, identifying and translating available guidelines.

Our method of categorising and scoring guidelines was binary, such that we scored a country’s guidelines as meeting a criterion, regardless of the frequency of instances where the criteria were met throughout the guidelines. To provide more differentiation, we highlighted where a criterion was met by only one statement for a country’s guidelines. However, we recognise that this approach does not provide a full understanding of how much information is being provided within guidelines and the depth and breadth of this information. Further research could include more fine-grained information of how well criteria are met as an additional measurement. This would also enable quantitative analysis in addition to content analysis. In addition, researchers could engage citizens and assess their interpretation of the guidelines in both quantitative and qualitative ways.

Whilst other reviews (e.g. [27]) analysed the language within guidelines, we did not include this in the current research. In their qualitative content analysis, [27] identified language within guidelines to determine countries’ position on plant-based diets. Further research could incorporate a sentiment analysis or similar into the current research methodology, to further contextualise information and recommendations. For example, Estonia’s guidelines recommend that ‘in the absence of a direct need, dairy products should not be replaced by so-called analogue products of plant origin’. We did not include this statement in analyses, as Estonia’s guidelines also contain a sustainability-motivated recommendation to reduce dairy intake. However, when considering both statements, one could argue that they are not fully recommending reducing dairy intake for sustainability. Bringing this element into the analysis would provide a richer understanding of a country’s position towards transitioning to more plant-based diets.

We did not consider a dimension relating to dietary swaps or substitutes in our analysis of written guidelines for their practical applicability, although we did include the ‘how’ component of the guidelines, which often included substitutions [27] and [37] explored substitutions explicitly in their research, and found that overall, few countries’ FBDG provide sufficient information about substituting animal-based foods for plant-based foods, especially when it comes to sustainability motivation. Further research could consider a substitutes/swaps dimension in addition to the other four dimensions we used in our analysis. Including this dimension could also be a useful avenue to explore the nutritional implications of reducing the intake of animal-based foods, particularly dairy, as we recognise that it can be an accessible and affordable source of key nutrients, such as protein, calcium, vitamin D. Furthermore, dairy foods are palatable and culturally accepted, whereas plant-based dairy alternatives are an emerging market, whose acceptability is varied. Therefore, it would be important to ensure appropriate messaging in guidelines about meeting nutritional needs and acceptable substitutes when substituting meat and dairy with plant-based foods to prevent nutrient deficiencies.

Future research could build upon the pictorial analysis we reported, for example combining this with the analyses of written guidelines instead of analysing both separately. Additionally, written guidelines or information alongside the pictures could be extracted, where possible, to contextualise the imagery and understand any health, sustainability or other justifications for recommendations. Within the current review, it is important to note that we deemed only a small number of countries’ guidelines eligible for the pictorial analysis. This may limit the interpretation or generalisability of the findings. Further research could compare all food guides regardless of sustainability and compare the findings for the guidelines that mention sustainability to those that do not. Additionally, future research could consider the cultural relevance and diversity of certain foods and food groups, to contextualise differences in proportions across countries’ food guides.

### Applied implications of guidelines: the need for change

We aimed to understand whether dietary guidelines contained recommendations to reduce meat and dairy consumption and whether this was justified by sustainability arguments, in addition to understanding the depth of these recommendations in explaining the what, why, how and quantities for dietary change. We found that most guidelines do not meet all criteria for the depth of recommendations; only five countries’ guidelines fulfilled the ‘why’ criterion (9% of all countries’ guidelines), and six fulfilled the ‘how’ (10% of all countries’ guidelines). Furthermore, whilst eight countries’ guidelines contained generic information about sustainability and diet, only six explicitly recommended reducing meat intake for sustainability and two for dairy. This indicates that guidelines are at risk of only providing vague and overarching information without being specific and actionable.

To enable dietary change, explicit sustainability-motivated information needs to accompany recommendations within guidelines. However, currently, sustainability is limited to side notes and suggestions within guidelines [29], and recommendations are often explicitly health-motivated or otherwise unclear [20,25]. Dietary guidance for health often has environmental sustainability co-benefits, such as a reduction in greenhouse gas emissions and land use, in addition to population health benefits of reduced all-cause mortality and all-cause cancer incidence rates [38]. However, nutrition information primarily motivated by health is not embedded in the wider context of sustainability [39]. The lack of this guidance could leave citizens feeling unequipped and unmotivated for dietary change, while population-level behaviour change is essential for the food systems change that is urgently needed. Notably, the dearth of actionable guidance with sustainability motivations gives the impression of governmental reluctance to include sustainability within FBDG [39]. It should be noted, however, that not all sustainable food choices will be healthier than less environmentally sustainable foods. Additionally, environmental sustainability is measured using GHG emissions, land use, and water use as the most widely used and compared metrics. The environmental impact of foods is relatively consistent across metrics. However, there are foods which measure differently depending on the metric. For example, nuts have an overall low environmental impact, however there is some evidence of higher water use and pesticide use in contrast to their associated lower land use and GHG emissions compared to other proteins [40,41]. Furthermore, differing farming practices, including those more specific to certain countries, can affect these measurements. Therefore, we encourage countries to be transparent about this in their guidelines and discuss the health and environmental impact of food choices to fully inform citizens.

Furthermore, the use of relative words such as ‘reduce’, ‘more’ and ‘higher intake’ within guidelines can make the guidance unclear and open to subjectivity. Previous research has recommended that guidance language be short, simple, specific and easy to understand [17], hence avoiding ambiguous language is important. Relative recommendations may refer to a country’s current average consumption, but this is not made explicit within guidelines. As individual consumption levels vary, it could be more useful to provide a quantified target or limit to accompany the relative recommendation. Then, an individual can make informed changes. Furthermore, citizens may struggle to visualise portions or weights of food as outlined in dietary guidelines [32]. Therefore, in addition to providing relative recommendations and quantities together for plant-based and animal-based foods, where possible it could prove beneficial for actionable guidance to include a visual guide for how to estimate portion sizes (such as in Ireland’s consumer-facing guidance, see OSF for details) or an example of an image of one serving (see [42] for the effects of pictorial serving size recommendations).

Little research so far has established how much people use or understand dietary guidelines. Citizens report low to average awareness of their country’s FBDG, with higher awareness of individual recommendations [43,44]. However, awareness and knowledge of guidelines are often far higher than dietary adherence [45]. Several factors affect how people interpret nutrition information. Existing or biased nutrition knowledge may lead to individuals dismissing nutrition information [46]. People’s trust in government nutritional guidance may be lower when it conflicts with media or market-delivered nutrition information [47]. Concerning sustainability, people underestimate the environmental impact of foods [48]. In the UK, citizens have a minimal understanding of food and sustainability and often question reducing meat and dairy intake as a sustainable initiative, favouring other popular initiatives, such as reducing single-plastic use or recycling [49].

Even when sustainability is incorporated into guidelines thoroughly, such as is the case for Sweden, the guidance assumes that individuals consider food and sustainability important and are ready to use the information to guide dietary choices [21]. It is, therefore, equally important to continue to examine how the public use and understand guidelines, and how to improve this through means such as government campaigns, the use of guidelines within food education, and the use of food guides as a form of nutrition intervention, considering both health and sustainability. Future research should establish the ways in which nutrition communication can most effectively affect eating behaviour, from both a health and sustainability perspective. Without these, we risk missing the opportunity of effectively using guidelines as a policy tool for nutrition communication.

In conclusion, this review demonstrates that very few countries include information and explicit recommendations to reduce meat and dairy intake for sustainability in FBDG. Furthermore, little consideration is given to how meat and dairy are represented as imagery alongside written guidelines. The transition towards sustainable diets requires a complex systems transformation, and will therefore require interdisciplinary collaboration including nutritionists, environmental scientists, economists and public health professionals. So, to not exacerbate existing health inequalities, accessibility and affordability of healthy, sustainable and culturally appropriate diets must be the focus of any such transition. Whilst we recognise the role of the broader drivers of the food system in dietary change and meeting climate change targets, guidelines play an important role in displaying dietary norms and acting as a tool to use in various settings, such as schools, hospitals, and public procurement more widely. Governments and relevant health authorities should make concerted efforts to rapidly update these guidelines consistently and efficiently with scientific evidence, and to ensure continued public understanding and use of guidelines through appropriate campaigns.

## Supplementary Material

Supplemental Material

## Data Availability

The data that support the findings of this study are openly available in the OSF, at http://doi.org/10.17605/OSF.IO/SQW48, reference number 10.17605/OSF.IO/SQW48.
