# Supplementary material for: Sustainability in food-based dietary guidelines: a review of recommendations around meat and dairy consumption and their visual representation
Source: Ann Med. 2025 Mar 7;57(1):2470252. doi: 10.1080/07853890.2025.2470252 (PMC11892039; doi:10.1080/07853890.2025.2470252)
Supplement: Supplemental Material [file IANN_A_2470252_SM3241.zip › Appendices/Appendix 1 - search strategy.docx]

**Appendix 1 – Search strategy**

**Information for search strategy**

If guidelines were irretrievable using the FAO database, we initially used links on the FAO website for a country’s relevant website, e.g., a health authority or government department. If a search function was available, we searched for “dietary guidelines” either on the English version of the site or we translated “dietary guidelines” into the target language, and searched for a FBDG document. If this was unsuccessful, we searched Google for using the following structure: (country name) AND (“dietary guidelines”) OR (“nutrition guidelines”) OR (“food based dietary guidelines”) AND (name if specified on FAO website). The first two pages of Google search results were used. We selected the most recent version if multiple documents or versions of documents were available. Standalone documents, e.g., PDFs, were chosen over live web pages; however, if only web pages were available, then these were selected. If documents were unavailable online and could only be ordered or were behind a paywall, these were deemed not publicly available and therefore excluded.
